# Supplementary material for: Measuring Italian citizens’ engagement in the first wave of the COVID-19 pandemic containment measures: A cross-sectional study
Source: PLoS One. 2020 Sep 11;15(9):e0238613. doi: 10.1371/journal.pone.0238613 (PMC7485890; doi:10.1371/journal.pone.0238613)
Supplement: S1 Appendix — Study survey–English version, (DOCX) [file pone.0238613.s001.docx]

**As you probably know, many people around the world have been diagnosed with a knew disease called COVID-19.**

**Recently, cases have been registered in Italy as well.**

**With the present survey, we ask you to tell us your recent experience, in particular regarding the consequences that the diffusion of the virus in Italy is having.**

**The results from this survey may contribute to the development of educative initiatives for citizens, to help them in this difficult times for public health.**

**We ask you to answer each of the following questions. There are not right or wrong answers, what really matters is your true personal experience.**

**The questionnaire will take about 10 minutes to complete.**

**We thank you for your cooperation.**

**The questionnaire is anonymous and the information will be treated according to the privacy regulations.**

**We ask for your consent to the processing of your personal and sensitive data pursuant to art. 13 of Legislative Decree 196/2003 and art. 13 GDPR 679/16 - "European regulation on the protection of personal data".**

**Do you provide your consent to the processing of your personal data?**

- **I AGREE**
- **I DO NOT GIVE CONSENT**

***How much are you worried for the COVID-19 emergency? (1=Not at all; 10=A lot)***

*1 2 3 4 5 6 7 8 9 10 I don’t know*

***In particular, how much do you feel at risk of being infected by the new Coronavirus? (1= Not at all 5 = A lot at risk).***

| ***Not at all*** | ***A little*** | ***Nor little nor much*** | ***Quite at risk*** | ***A lot at risk*** |  | ***I don’t have an opinion*** |
| --- | --- | --- | --- | --- | --- | --- |
| ***1*** | ***2*** | ***3*** | ***4*** | ***5*** |  | ***6*** |

***Following, you’ll find 5 statements that describe how a person can feel when thinking about the risk of being infected from the new Coronavirus (COVID-19). Each sentence can be completed by choosing one of the 4 specific states, or the intermediate points between the different states. Please, indicate the position that best indicates your state, by clicking on the corresponding dot.***

***Please, check that you have answered all the statements and that you have indicated only one option for each of them.***

| *Thinking about the COVID-19 pandermic, you feel...* | | | | | | | |  |
| --- | --- | --- | --- | --- | --- | --- | --- | --- |
|  |  |  |  |  |  |  |  |  |
| *1* | I feel like I’m in blackout  O | O | I feel in alert  O | O | I feel informed  O | O | I feel positive  O |  |
| *2* | I feel lost  O | O | I feel alarmed  O | O | I feel conscious  O | O | I feel in peace  O |  |
| *3* | I feel overwhelmed by emotions  O | O | I feel in anxiety every time I hear talking about the Covid-19  O | O | I feel I got used to this emergency  O | O | My life goes on regardless of this situation  O |  |
| *4* | I'm living moments of great discouragement  O | O | I often feel anxious  O | O | I feel I got used to this situation  O | O | I am generally optimist about my future and my health  O |  |
| *5* | I feel paniked  O | O | I feel the urgency to do something  O | O | I keep calm  O | O | I am in control  O |  |

***Now please indicate your degree of agreement / disagreement with the statements below on the new Coronavirus infection (COVID-19) indicating the option that best reflects your current experience.***

|  | ***Completely disagree*** | ***Disagree*** | ***Nor agree nor disagree*** | ***Agree*** | ***Completely agree*** |
| --- | --- | --- | --- | --- | --- |
| I have the primary responsibility in preventing the infection by Covid-19 |  |  |  |  |  |
| I think that the emergency regarding Covid-19 has been created by an overblown mass-media hype |  |  |  |  |  |

***Now please indicate your degree of agreement / disagreement with the statements below on health management, indicating the option that best reflects your current experience.***

|  | ***Completely disagree*** | ***Disagree*** | ***Nor agree nor disagree*** | ***Agree*** | ***Completely agree*** |
| --- | --- | --- | --- | --- | --- |
| I can manage my own health effectively |  |  |  |  |  |
| I can manage my own health even when I’m distressed |  |  |  |  |  |
| it’s important to cooperate with healthcare professionals in defining how to manage my own health |  |  |  |  |  |
| I fully trust scientific research |  |  |  |  |  |
| I fully trust the National Healthcare System |  |  |  |  |  |

**In the last week, how often have you used the following information channels to search for information about Coronavirus (COVID-19)? (1 = Never, 2 = Rarely, 3 = Sometimes, 4 = Often, 5=More than once a day)**

|  | 1  Never | 2  Rarely | 3  Sometimes | 4 Usually | 5  More than once a day |
| --- | --- | --- | --- | --- | --- |
| News |  |  |  |  |  |
| Television programs |  |  |  |  |  |
| Radio |  |  |  |  |  |
| Web sites |  |  |  |  |  |
| Social Network |  |  |  |  |  |
| Specialized magazines |  |  |  |  |  |
| Newspapers |  |  |  |  |  |
| Scientific magazines |  |  |  |  |  |
| Family doctor |  |  |  |  |  |
| Number for Coronavirus |  |  |  |  |  |
| Others |  |  |  |  |  |

***On February, 21st, the news of an Italian patient infected by the new Coronavirus COVID-19 in the area of Codogno (Lo) was diffused. After hearing this news, have you increased or decreased any of the following purchases/consumption in your family?...***

|  | **diminished** | **unchanged** | **increased** | **I don't usually buy** |
| --- | --- | --- | --- | --- |
| Fresh food products |  |  |  |  |
| Frozen food products |  |  |  |  |
| Canned food products |  |  |  |  |
| Personal care products |  |  |  |  |
| Products for personal disinfection |  |  |  |  |
| Household disinfection products |  |  |  |  |

***And after becoming aware of the same news, did you adopt the following behaviors?***

|  | yes | no |
| --- | --- | --- |
| have you reduced meals in restaurants |  |  |
| have you reduced meals in ethnic restaurants |  |  |

***Today, would you be willing to buy food products coming from “red” zones (Lodigiano, cremonese…)?***

- - Yes
  - No
  - I don’t know

***In the last week* have you been stockpiling food and first need products?**

- - Yes
  - No

**The questionnaire is almost over, we’ll now ask a few information about yourself**

**Gender**

- - Male
  - Female

**Year of birth (in numbers)**

|  |
| --- |

**Region of residence**

- - North-West
  - North-East
  - Center
  - South and Islands

**Inhabited centre size**

- - Up to 10000 inhabitants
  - 10001/100000 inhabitants
  - 100001/500.000 inhabitants
  - More than 500.000 inhabitants
  - I don’t know

**Employment**

- Entrepreneur / freelancer
- Manager / official / middle manager
- Employee / teacher / military
- Worker / shop assistant / apprentice
- Housewife
- Student
- Retired
- Unoccupied
- Other

**Level of education**

- Middle school or lower
- High school
- University degree

**Are you suffering from one or more chronic diseases?**

- - Yes
  - no
