# Supplementary material for: Measuring Italian citizens’ engagement in the first wave of the COVID-19 pandemic containment measures: A cross-sectional study
Source: PLoS One. 2020 Sep 11;15(9):e0238613. doi: 10.1371/journal.pone.0238613 (PMC7485890; doi:10.1371/journal.pone.0238613)
Supplement: S2 Appendix — Study survey–Italian version. (DOCX) [file pone.0238613.s002.docx]

**Come saprà, molte persone nel mondo sono state recentemente diagnosticate con una nuova malattia chiamata nuovo Coronavirus (COVID-19)**

**Recentemente nuovi casi di contagio sono stati segnalati anche in Italia.**

**Con questo questionario le chiediamo di raccontarci la sua esperienza di queste ore, con particolare riferimento alle ricadute che la diffusione del virus in Italia sta avendo sul suo stile di vita.**

**I risultati di questa indagine potranno contribuire all’orientamento di iniziative di comunicazione e sensibilizzazione rivolte ai cittadini al fine di meglio sostenerli in queste ore complesse per la salute pubblica.**

**Le chiediamo di rispondere a ciascuna delle domande che seguono. Non ci sono risposte giuste o sbagliate, quello che conta è la sua esperienza personale.**

**Il questionario richiederà circa 10 minuti per la compilazione. 
La ringraziamo per la collaborazione.**

**Il questionario è anonimo e le informazioni saranno trattate secondo le normative della privacy.

Le chiediamo il consenso al trattamento dei tuoi dati personali e sensibili ai sensi dell’art.13 del D.Lgs.196/2003 e dell’art. 13 GDPR 679/16 – “Regolamento europeo sulla protezione dei dati personali”.

Fornisce il Suo consenso al trattamento dei suoi dati personali?**

- DO IL CONSENSO
- NON DO IL CONSENSO

***Lei quanto si direbbe preoccupato per l’emergenza Coronavirus (COVID-19)? (1= per nulla preoccupato, 10 = molto preoccupato)***

*1 2 3 4 5 6 7 8 9 10 Non saprei*

***Più in particolare, quanto si sente a rischio di contagio dal nuovo Coronavirus (COVID-19) su una scala da 1 a 5 (1=per niente a rischio; 5 = Molto a rischio).***

| ***Per niente a rischio*** | ***Poco a rischio*** | ***Né poco né molto a rischio*** | ***Abbastanza a rischio*** | ***Molto a rischio*** |  | ***Non ho un’opinione*** |
| --- | --- | --- | --- | --- | --- | --- |
| ***1*** | ***2*** | ***3*** | ***4*** | ***5*** |  | ***6*** |

***Qui di seguito trova 5 affermazioni che descrivono come una persona può sentirsi pensando al rischio di contagio dal nuovo Coronavirus (COVID-19). Ciascuna frase può essere completata scegliendo uno dei 4 stati specifici, oppure i punti intermedi fra i diversi stati. Le chiediamo di indicare la sua posizione rispetto allo stato che più la rispecchia, cliccando sul pallino corrispondente.***

***La preghiamo di controllare di aver risposto a tutte le affermazioni e di aver indicato solo una opzione per ognuna di esse.***

| *Pensando all’epidemia del Coronavirus…* | | | | | | | |
| --- | --- | --- | --- | --- | --- | --- | --- |
|  |  |  |  |  |  |  |  |
| *1* | Mi sembra di essere in blackout  O | O | Mi sento in allerta  O | O | Mi sento consapevole  O | O | Mi sento positivo/a  O |
| *2* | Mi sento perduto  O | O | Mi sento in allarme  O | O | Sono cosciente  O | O | Mi sento sereno/a  O |
| *3* | Mi sento sopraffatto dalle emozioni  O | O | Sono in ansia ogni volta che sento parlare di Coronavirus  O | O | Sento di essermi abituato a questa emergenza  O | O | La mia vita va avanti lo stesso nonostante questa situazione  O |
| *4* | Vivo momenti di grande sconforto  O | O | Mi sento  spesso in ansia  O | O | Sento di essermi adattato a questa situazione  O | O | Sono tendenzialmente ottimista sul mio futuro e il mio stato di salute  O |
| *5* | Sono nel panico  O | O | Sento l’urgenza di fare qualcosa  O | O | Cerco di mantenere la calma  O | O | Sento di  avere il controllo  O |

***Le chiediamo ora di indicare il suo grado di accordo/disaccordo con le affermazioni riportate qui di seguito in merito al contagio da nuovo Coronavirus (COVID-19) indicando l’opzione che più rispecchia la sua attuale esperienza.***

|  | ***Molto in disaccordo*** | ***In disaccordo*** | ***Né in accordo né in disaccordo*** | ***In accordo*** | ***Molto in accordo*** |
| --- | --- | --- | --- | --- | --- |
| Sono io il primo responsabile nel prevenire il mio rischio di contagio da nuovo Coronavirus (COVID-19) |  |  |  |  |  |
| Penso che l’allarme nuovo Coronavirus (COVID-19) sia il frutto di una montatura mediatica |  |  |  |  |  |

***Le chiediamo ora di indicare il suo grado di accordo/disaccordo con le affermazioni riportate qui di seguito in merito alla gestione della salute indicando l’opzione che più rispecchia la sua attuale esperienza.***

|  | ***Molto in disaccordo*** | ***In disaccordo*** | ***Né in accordo né in disaccordo*** | ***In accordo*** | ***Molto in accordo*** |
| --- | --- | --- | --- | --- | --- |
| So gestire la mia salute in modo efficace |  |  |  |  |  |
| Anche se sotto stress, riesco a occuparmi della mia salute |  |  |  |  |  |
| È importante collaborare con il persole sanitario nel definire come gestire la propria salute |  |  |  |  |  |
| Ho piena fiducia nella ricerca scientifica |  |  |  |  |  |
| Ho piena fiducia nel sistema sanitario |  |  |  |  |  |
| Ho piena fiducia nelle istituzioni |  |  |  |  |  |

**Nell’ultima settimana con quale frequenza ha utilizzato i seguenti canali informativi per documentarsi sul Coronavirus *(COVID-19)*? *(1 = Mai, 2 = Raramente, 3 = Occasionalmente*, 4 = Spesso, *5 = Più di una volta al giorno*)**

|  | 1  (Mai) | 2  (Raramente) | 3  (Occasionalmente) | 4  (Spesso) | 5  (Più di una volta al giorno) |
| --- | --- | --- | --- | --- | --- |
| Telegionali |  |  |  |  |  |
| Programmi televisivi di approfondimento (es. puntate di approfondimento, interviste a medici, ) |  |  |  |  |  |
| Radio |  |  |  |  |  |
| Siti Web |  |  |  |  |  |
| Social Network |  |  |  |  |  |
| Riviste specializzate |  |  |  |  |  |
| Quotidiani |  |  |  |  |  |
| Riviste scientifiche |  |  |  |  |  |
| Medico di famiglia |  |  |  |  |  |
| Numero Verde per il Coronavirus |  |  |  |  |  |

Altro (aperto)

***Venerdì 21 febbraio si è diffusa la notizia di un paziente italiano contagiato dal nuovo Coronavirus COVID-19 nella zona di Codogno (Lo). Dopo essere venuto a conoscenza di questa notizia, nella Sua famiglia avete aumentato o diminuito qualcuno dei seguenti acquisti/consumi?….***

|  | **Diminuito** | **Rimasto uguale** | **Aumentato** | **Non sono solito acquistarlo** |
| --- | --- | --- | --- | --- |
| Prodotti alimentari freschi |  |  |  |  |
| Prodotti alimentari surgelati |  |  |  |  |
| Prodotti alimentari in scatola/lattina |  |  |  |  |
| Prodotti per la cura personale |  |  |  |  |
| Prodotti per la disinfezione personale |  |  |  |  |
| Prodotti per la disinfezione della casa |  |  |  |  |

***E dopo l’essere venuto a conoscenza della medesima notizia le è capitato di adottare i seguenti comportamenti?***

|  | si | no |
| --- | --- | --- |
| Ho ridotto i pasti fuori casa |  |  |
| Ho ridotto i pasti in ristoranti etnici |  |  |

***E oggi come oggi, acquisterebbe prodotti alimentari provenienti dalle zone “focolaio” (cremonese, lodigiano…)?***

- - Si
  - No
  - Non so

***Nell’ultima settimana, lei ha accumulato scorte alimentari e beni di prima necessità?***

- - Si
  - No

***Il questionario è quasi giunto al termine. Per finire, le chiediamo alcune informazioni su di lei.***

**Genere**

- - Uomo
  - Donna

**Anno di nascita (in cifre)**

|  |
| --- |

**Regione di residenza**

- - Nord-ovest
  - Nord-est
  - Centro
  - Sud e isole

**Ampiezza demografica del comune di residenza**

- - Fino a 10.000 abitanti
  - 10.001/100.000 abitanti
  - 100.001/500.000 abitanti
  - Più di 500.000 abitanti
  - Non saprei

**Occupazione**

- - Imprenditore/freelancer
  - Manager/ufficiale
  - Impiegato/insegnante/militare
  - Operaio/commesso/apprendista
  - Casalinga/o
  - Studente
  - Pensionato
  - Disoccupato
  - Altro

**Titolo di studio**

- - Scuole medie o meno
  - Scuole superiori
  - Laurea

**È affetto da una o più malattie croniche**

- - si
  - no
