# Supplementary material for: Measuring Italian citizens’ engagement in the first wave of the COVID-19 pandemic containment measures: A cross-sectional study
Source: PLoS One. 2020 Sep 11;15(9):e0238613. doi: 10.1371/journal.pone.0238613 (PMC7485890; doi:10.1371/journal.pone.0238613)
Supplement: S3 Appendix — (PDF) [file pone.0238613.s003.pdf]

UNIVERSITÀ CATTOLICA DEL SACRO CUORE

20123 MILANO – LARGO A. GEMELLI 1

Dipartimento di Psicologia

Università Cattolica del Sacro Cuore

Commissione Etica per la Ricerca in Psicologia (CERPS)

Dichiarazione di Approvazione della Commissione etica - CERPS

Titolo della ricerca: **Monitoraggio sui consumi alimentari degli italiani in una prospettiva di psicologia dei consumi**

Proponenti: Guendalina Graffigna
Altri proponenti: Maria Grazia Savarese

data di sottomissione: 30 ottobre 2019
data di approvazione: 15 gennaio 2020
numero protocollo: 02-20

In merito allo studio in oggetto, la commissione etica ha valutato i seguenti documenti:

- ***Modulo richiesta***
- ***Questionario***
- ***Consenso informato***

La commissione etica ha espresso all'unanimità parere favorevole allo studio

Si produce la seguente per gli adempimenti del caso, secondo le rispettive competenze

Il Presidente del CERPS

Prof. Camillo Regalia

Direttore del Dipartimento di Psicologia

Prof.Alessandro Antonietti

Milano 22 giugno 2020
