# Supplementary material for: Measuring Italian citizens’ engagement in the first wave of the COVID-19 pandemic containment measures: A cross-sectional study
Source: PLoS One. 2020 Sep 11;15(9):e0238613. doi: 10.1371/journal.pone.0238613 (PMC7485890; doi:10.1371/journal.pone.0238613)
Supplement: Attachment — Submitted filename: Response to reviewers.docx [file pone.0238613.s006.docx]

**Comments from the editors and reviewer**

We are very grateful to have been given the opportunity to revise our manuscript for *Plos One.*

We thank the referee and editor for their comments to strengthen the presentation of our work. We have modified the text to respond to all the issues and have elaborated on the changes below.

**Comment 1**

In table1, chronic patient contained what types of disease and also the YES or No respectively represent the proportion of men and women or the proportion of chronic diseases in the total population?

*Response*:

Thanks for your comments. In our survey we asked participants whether they do or not suffer from a chronic disease; hence, the table 1 simply reports frequencies of responses (Yes vs No) in our sample, we have no means to know the types of diseases of which they suffer. We changed the label for more clarity.

**Comment 2**
In table3, the item was HE1,2,3,4,5,what does these mean?

*Response*:

Thanks for this comment. It means “health engagement”, which is the measured variable. We modified the label for more clarity.

**Comment 3**
In the part results, which contains too many methods, so please revise and reorganize the results and methods.

*Response*:

Thanks. We revised the two sections moving some part from the results to the method section.

**Comment 4**
The following references should be discussed in the revision text.
Cao JL, Hu XR, Tu WJ., & Liu Q. (2020). Clinical Features and Short-term Outcomes of 18 Patients with Corona Virus Disease 2019 in Intensive Care Unit. Intensive Care Medicine, DOI: 10.1007/s00134-020- 05987-7.
Cao JL, Tu WJ, Hu XR, & Liu Q. (2020). Clinical Features and Short-term Outcomes of 102 Patients with Corona Virus Disease 2019 in Wuhan,China. Clinical Infectious Diseases, DOI: 10.1093/cid/ciaa243/ 5814897.

*Response*:

Thanks for this suggestion. We added this reference in the introduction section.

**Comment 5**
While the authors stated the sample was representative, they did not provide comparison to the Italian adult population data, and the representativeness of the sample remains questionable, So on behalf of the people who participated in the research, how to explain the reliability of the experiment

*Response*:

The sample was representative of the Italian population due to the sampling method adopted, we’ve added further details in the methods section. Comparison data are provided in Table 1 under the “% Italian population” columns and retrieved from the website of ISTAT (the Italian National Institute of Statistics). Therefore, we assume that the study results are valid for not only for the sample of the study, but also for the larger Italian Population. Surely, further study on other sample should confirm that. We stressed this aspect in the limitation section.
